# (E)-4-(3-(3,5-dimethoxyphenyl)allyl)-2-methoxyphenol inhibits growth of colon tumors in mice

**DOI:** 10.18632/oncotarget.5861

**Published:** 2015-10-14

**Authors:** Jie Zheng, Mi Hee Park, Dong Ju Son, Min Gi Choi, Jeong Soon Choi, Kyung Tak Nam, Hae Deun Kim, Kevin Rodriguez, Benjamin Gann, Young Wan Ham, Sang Bae Han, Jin Tae Hong

**Affiliations:** ^1^ College of Pharmacy and Medical Research Center, Chungbuk National University, Heungduk-gu, Cheongju, Chungbuk, Republic of Korea; ^2^ Department of Chemistry, Utah Valley University, Orem, UT, USA

**Keywords:** colon cancer, apoptosis, STAT3, NF-κB, death receptor

## Abstract

In our previous study, we found that (E)-2,4-bis(p-hydroxyphenyl)-2-butenal showed anti-cancer effect, but it showed lack of stability and drug likeness. We have prepared several (E)-2,4-bis(p-hydroxyphenyl)-2-butenal analogues by Heck reaction. We selected two compounds which showed significant inhibitory effect of colon cancer cell growth. Thus, we evaluated the anti-cancer effects and possible mechanisms of one compound (E)-4-(3-(3,5-dimethoxyphenyl)allyl)-2-methoxyphenol *in vitro* and *in vivo*. In this study, we found that (E)-4-(3-(3,5-dimethoxyphenyl)allyl)-2-methoxyphenol induced apoptotic cell death in a dose dependent manner (0-15 μg/ml) through activation of Fas and death receptor (DR) 3 in HCT116 and SW480 colon cancer cell lines. Moreover, the combination treatment with (E)-4-(3-(3,5-dimethoxyphenyl)allyl)-2-methoxyphenol and nuclear factor κB (NF-κB) inhibitor, phenylarsine oxide (0.1 μM) or signal transducer and activator of transcription 3 (STAT3) inhibitor, Stattic (50 μM) increased the expression of Fas and DR3 more significantly. In addition, (E)-4-(3-(3,5-dimethoxyphenyl)allyl)-2-methoxyphenol suppressed the DNA binding activity of both STAT3 and NF-κB. Knock down of STAT3 or NF-κB p50 subunit by STAT3 small interfering RNA (siRNA) or p50 siRNA magnified (E)-4-(3-(3,5-dimethoxyphenyl)allyl)-2-methoxyphenol-induced inhibitory effect on colon cancer cell growth. Besides, the expression of Fas and DR3 was increased in STAT3 siRNA or p50 siRNA transfected cells. Moreover, docking model and pull-down assay showed that (E)-4-(3-(3,5-dimethoxyphenyl)allyl)-2-methoxyphenol directly bound to STAT3 and NF-κB p50 subunit. Furthermore, (E)-4-(3-(3,5-dimethoxyphenyl)allyl)-2-methoxyphenol inhibited colon tumor growth in a dose dependent manner (2.5 mg/kg-5 mg/kg) in mice. Therefore, these findings indicated that (E)-4-(3-(3,5-dimethoxyphenyl)allyl)-2-methoxyphenol may be a promising anti-cancer agent for colon cancer with more advanced research.

## INTRODUCTION

Colorectal cancer (CRC, also known as colon cancer, bowel cancer) ranks third among the leading causes of cancer-associated death after lung and prostate cancer for men and after lung and breast cancer for women [1]. On the other hand, colon cancer is also one of the most curable cancers if it is detected in early stage through regular colonoscopy [2]. Systemic chemotherapy plays an integral part in advanced colon cancer treatments, however, 50% of patients respond poorly or have disease progression due to resistance to chemotherapeutic agents [3]. As present treatments for colon cancer patients are not so sufficient, it is urgent to develop appropriate novel chemo-preventive compounds.

Apoptosis is the process of programmed cell death which has an important role in anti-cancer effects of chemotherapeutics [4]. Activated death receptors (DRs) induce apoptosis through caspase activation [5]. DRs are activated by binding to their ligands (interaction of DR1 with TNF; Fas with FasL; DR3 with TWEAK; DR4 and DR5 with TRAIL; Ligand of DR6 has not been exactly defined) [6, 7]. Activation of death receptors induces activation of caspase-8, which leads to the activation of downstream caspases, including caspase-9 and caspase-3, as well as the translocation of Bax to mitochondria leading to apoptosis [8]. Increase of death receptor expression could enhance susceptibility of cancer cells toward chemotherapeutics [9].

Signal Transducer and Activator of Transcription 3 (STAT3) belongs to the STAT family of proteins, which are both signal transducers and transcription factors [10]. STAT3 is a key signal transduction protein that mediates signaling by many cytokines, hormones, growth factors, and oncoproteins [11]. Once theses ligands bind to the specific transmembrane STAT3 receptor, STAT3 becomes activated by tyrosine phosphorylation and dimerizes through reciprocal Src homology 2-phosphotyrosine binding, and the dimeric STAT3 translocates to the nucleus, where it binds to consensus STAT3 binding sequences within the promoter region of target genes and thereby activates their transcription [11]. Phosphorylation of STAT3 performs a vital function in cell growth, proliferation, survival, differentiation, apoptosis, metastasis and angiogenesis [14-17]. Constitutively activated STAT3 has been identified in many cancers including colon cancer [18]. Studies in the past few years have provide compelling evidence for the critical role of aberrant STAT3 in malignant transformation and tumorigenesis, thus, it is now generally accepted that STAT3 is one of the critical players in human cancer formation and represents a valid target for novel anti-cancer drug design [10]. Selected natural inhibitors of the STAT3 signaling pathway are Betulinic acid, Butein, Caffeic acid, Capsaicin, Celastrol, Cucurbitacins, Curcumin, Diosgenin, Guggulsterone, Honokiol and so on [13]. As STAT3 is activated by dimerization and then binds DNA to perform its functions, specific inhibitors targeting the disruption of their protein-protein binding or DNA-binding activity, are more promising agents [13].

On the other hand, drugs aimed at multiple pathways can be more efficacious and less vulnerable to acquire resistance because the disease system is less able to compensate for the action of two or more drugs simultaneously, and this approach can be particularly beneficial in cancers because oncogenesis is known to be a multistep process [13, 19]. NF-κB is constitutively activated in human colorectal carcinoma tissue and colon cancer cells [20]. NF-κB plays a crucial role in the suppression of apoptosis as well as in the induction of cell proliferation and inflammation, NF-κB is closely associated with cancer development [21]. NF-κB acts as a cell survival factor through its regulatory role in the expression of an array of apoptotic (caspase-3 and Bax), antiapoptotic (Bcl-2 and IAP family), and cell proliferation genes (cyclooxygenase-2 and cyclins) [22]. NF-κB and STAT3 are rapidly activated in response to various stimuli including stresses and cytokines, although they are activated by entirely different signaling mechanisms. Once activated, NF-κB and STAT3 control the expression of anti-apoptotic, pro-proliferative and immune response genes some of which overlap and require transcriptional cooperation between the two factors [23]. Therefore, inhibition of NF-κB and STAT3 by chemotherapeutics is intended as a potential strategy to eliminate cancerous cells through induction of apoptosis.

Our previous study showed that (E)-2,4-bis(p-hydroxyphenyl)-2-butenal has anti-cancer activity [24]. Recently, to enhance the stability and drug likeness of (E)-2,4-bis(p-hydroxyphenyl)-2-butenal, we synthesized (E)-4-(3-(3,5-dimethoxyphenyl)allyl)-2-methoxyphenol by Heck reaction, and we found that this compound has more stable and drug likeness properties. Therefore, in this study we investigated the anti-cancer effects and possible mechanisms of (E)-4-(3-(3,5-dimethoxyphenyl)allyl)-2-methoxyphenol on colon cancer cell growth *in vivo* and *in vitro*.

## RESULTS

### Effect of (E)-4-(3-(3,5-dimethoxyphenyl)allyl)-2-methoxyphenol on the growth of colon cancer cells

**Figure 1:**
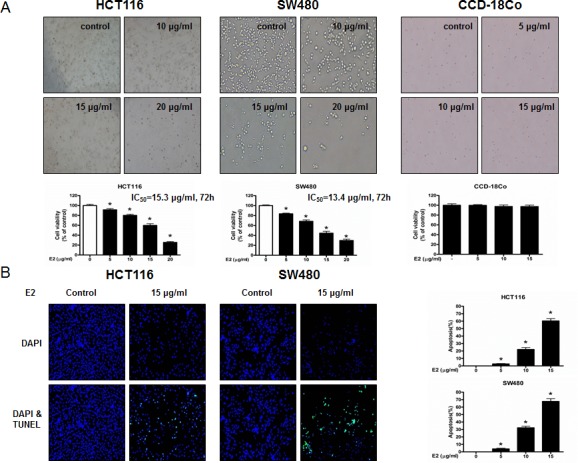
Effect of (E)-4-(3-(3,5-dimethoxyphenyl)allyl)-2-methoxyphenol on cell growth and apoptotic cell death in colon cancer cells **A.** (E)-4-(3-(3,5-dimethoxyphenyl)allyl)-2-methoxyphenol treatment induces cell death in HCT116 and SW480 colon cancer cells but not in CCD-18Co normal cells. Relative cell survival rate was determined by MTT assay. **B.** Apoptotic cell death of HCT116 and SW480. Colon cancer cells were treated with (E)-4-(3-(3,5-dimethoxyphenyl)allyl)-2-methoxyphenol (0-15 μg/ml) for 24 h, and then labeled with DAPI and TUNEL solution. Total number of cells in a given area was determined by using DAPI nuclear staining (fluorescent microscope). A green color in the fixed cells marks TUNEL-labeled cells. Apoptotic index was determined as the DAPI-stained TUNEL-positive cell number / total DAPI-stained cell number × 100%. Data was expressed as the mean ± S.D. of three experiments. *p < 0.05 indicates significant difference from control group.

To evaluate the effect of (E)-4-(3-(3,5-dimethoxyphenyl)allyl)-2-methoxyphenol on the cell growth of colon cancer cells, we analyzed cell proliferation assay. (E)-4-(3-(3,5-dimethoxyphenyl)allyl)-2-methoxyphenol (0-20 μg/ml) induced cell death in colon cancer cells but not normal cells (Figure 1A). IC_50_ values of HCT116 and SW480 (Figure 1A) were 15.3 and 13.4 μg/ml, respectively. To determine whether the inhibition of cell growth by the (E)-4-(3-(3,5-dimethoxyphenyl)allyl)-2-methoxyphenol was due to the induction of apoptosis, we evaluated the changes in colon cancer cells by using 4,6-diamidino-2-phenylindole (DAPI) staining followed by TdT-mediated dUTP nick and labeling (TUNEL) assays, and then the double labeled cells were analyzed by fluorescence microscope. The cells were treated with concentrations of (E)-4-(3-(3,5-dimethoxyphenyl)allyl)-2-methoxyphenol (0-15 μg/ml) for 24 h. DAPI-stained TUNEL-positive cells were concentration-dependently increased and the highest concentration of (E)-4-(3-(3,5-dimethoxyphenyl)allyl)-2-methoxyphenol (15 μg/ml) caused most of cells TUNEL-positive, and apoptosis rates were 60.31% in HCT116 cells and 67.64% in SW480 cells (Figure 1B). These results demonstrated that (E)-4-(3-(3,5-dimethoxyphenyl)allyl)-2-methoxyphenol strongly induced apoptosis in colon cancer cells.

### Effect of (E)-4-(3-(3,5-dimethoxyphenyl)allyl)-2-methoxyphenol on the expression of apoptosis regulatory proteins

**Figure 2:**
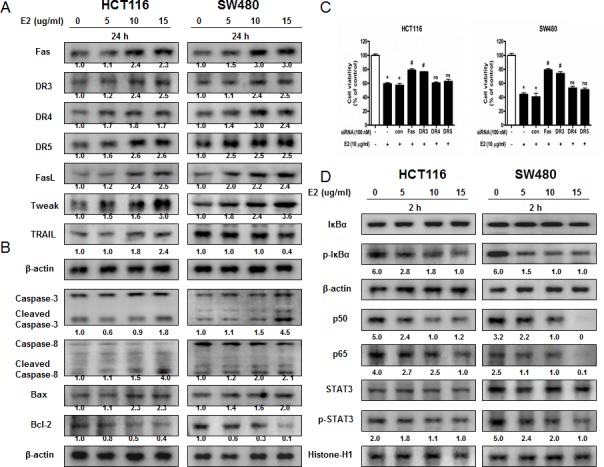
Effect of (E)-4-(3-(3,5-dimethoxyphenyl)allyl)-2-methoxyphenol on the expression of apoptosis regulatory proteins and on the expression of NF-κB and STAT3 **A.** Expression of apoptosis regulatory proteins related extrinsic pathway was determined by Western blot analysis with antibodies against Fas, DR3, DR4, DR5, FasL, TWEAK, TRAIL and β-actin (internal control). **B.** Intrinsic pathway was determined by Western blot analysis with antibodies against cleaved capase-3, cleaved caspase-8, Bax, Bcl-2 and β-actin (internal control). Each band is representative for three experiments. **C.** Effect of DRs on the inhibitory effect of (E)-4-(3-(3,5-dimethoxyphenyl)allyl)-2-methoxyphenol in colon cancer cells. Relative cell survival rate was determined by MTT assay. **D.** Cytosolic proteins were used to determine expression of IκB, p-IκBα and β-actin (internal control) and nuclear proteins were used to determine expression of STAT3, p-STAT3, p50, p65 and Histone H1 (internal control) in colon cancer cells. Each band is representative for three experiments.

To figure out the relationship between the induction of apoptosis and the expression of their regulatory proteins by (E)-4-(3-(3,5-dimethoxyphenyl)allyl)-2-methoxyphenol, the expression of apoptosis related extrinsic pathway (Figure 2A) and intrinsic pathway (Figure 2B) proteins was investigated. (E)-4-(3-(3,5-dimethoxyphenyl)allyl)-2-methoxyphenol treatment increased the expression of various apoptotic proteins such as Bax, cleaved caspase-3, cleaved caspase-8 as well as the expression of death receptors like Fas and DR3 in a concentration dependent manner (0-15 μg/ml). However, the expression of anti-apoptotic protein Bcl-2 was decreased. To investigate the effect of knock down of DRs on the (E)-4-(3-(3,5-dimethoxyphenyl)allyl)-2-methoxyphenol induced cell death, we performed transfection experiment with siRNA. As a result, knock down of Fas or DR3 partially reversed the inhibitory effect of (E)-4-(3-(3,5-dimethoxyphenyl)allyl)-2-methoxyphenol on colon cancer cells but not knock down of DR4 or DR5 (Figure 2D).

### Effect of (E)-4-(3-(3,5-dimethoxyphenyl)allyl)-2-methoxyphenol on STAT3 activation

**Figure 3:**
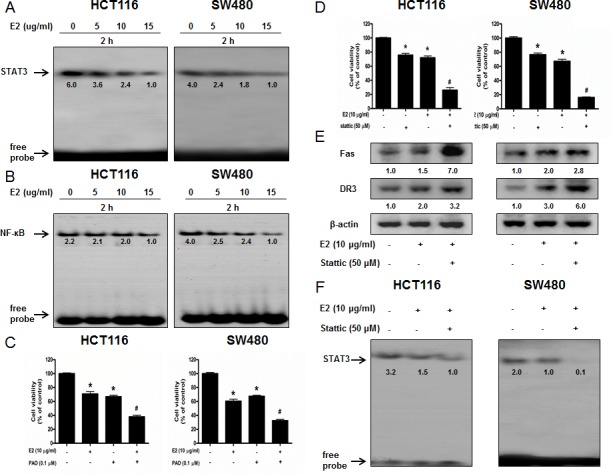
Effect of (E)-4-(3-(3,5-dimethoxyphenyl)allyl)-2-methoxyphenol on the DNA binding activity of NF-κB and STAT3 and ffect of STAT3 inhibitor and NF-κB inhibitor on (E)-4-(3-(3,5-dimethoxyphenyl)allyl)-2-methoxyphenol-induced colon cancer cell growth **A.** & **B.** Colon cancer cells were treated with (E)-4-(3-(3,5-dimethoxyphenyl)allyl)-2-methoxyphenol (0-15 μg/ml) for 2 h, and then were lysed. Nuclear extract was incubated in binding reactions of ³²p-end-labeled oligo nucleotide containing the STAT3 or NF-κB sequence. The present EMSA results are representative for three experiments. **C.** & **D.** Cells were pretreated with NF-κB inhibitor PAO (0.1 μM) or STAT3 inhibitor Stattic (50 μM) for 1 h and then were treated with (E)-4-(3-(3,5-dimethoxyphenyl)allyl)-2-methoxyphenol for 24 h. Relative cell survival rate was determined by MTT assay. **E.** Whole cell extracts were analyzed by Western blotting using Fas, DR3 and β-actin antibodies. **F.** Effect of STAT3 inhibitor on the DNA binding activity of STAT3. Each band is representative for three experiments. *p < 0.05, indicates significant difference from control cells. #*p* < 0.05 indicates significant difference from (E)-4-(3-(3,5-dimethoxyphenyl)allyl)-2-methoxyphenol-treated cells.

STAT3 plays a vital role in colon cancer cell growth. To investigate whether (E)-4-(3-(3,5-dimethoxyphenyl)allyl)-2-methoxyphenol inactivates STAT3, we performed electro mobility shift assay (EMSA) for detecting DNA binding activity of STAT3. We found that (E)-4-(3-(3,5-dimethoxyphenyl)allyl)-2-methoxyphenol untreated colon cancer cells showed highly constituted activation of STAT3 in both colon cancer cells. However, the treatment of (E)-4-(3-(3,5-dimethoxyphenyl)allyl)-2-methoxyphenol concentration dependently inhibited DNA binding activity of STAT3 (Figure 3A). Agreed with the inhibition of STAT3, nucleus translocation of phosphorylation of STAT3 was inhibited by (E)-4-(3-(3,5-dimethoxyphenyl)allyl)-2-methoxyphenol in both colon cancer cells (Figure 2E).

### Effect of (E)-4-(3-(3,5-dimethoxyphenyl)allyl)-2-methoxyphenol on NF-κB activation

NF-κB also plays a vital role in colon cancer cell growth. To investigate whether (E)-4-(3-(3,5-dimethoxyphenyl)allyl)-2-methoxyphenol inactivates NF-κB, we performed EMSA for detecting DNA binding activity of NF-κB. We found that (E)-4-(3-(3,5-dimethoxyphenyl)allyl)-2-methoxyphenol untreated colon cancer cells showed highly constituted activation of NF-κB in both colon cancer cells. However, the treatment of (E)-4-(3-(3,5-dimethoxyphenyl)allyl)-2-methoxyphenol concentration dependently inhibited DNA binding activity of NF-κB (Figure 3B). Agreed with the inhibition of NF-κB, cytosolic phosphorylation of IκB as well as the nucleus translocation of p50 and p65 were inhibited by (E)-4-(3-(3,5-dimethoxyphenyl)allyl)-2-methoxyphenol in both colon cancer cells (Figure 2E).

### Combination effect of (E)-4-(3-(3,5-dimethoxyphenyl)allyl)-2-methoxyphenol and STAT3 inhibitor on the growth of human colon cancer cells

To further investigate whether STAT3 and NF-κB play a critical role in (E)-4-(3-(3,5-dimethoxyphenyl)allyl)-2-methoxyphenol-induced activation of death receptors as well as inhibition of colon cancer cell growth, we pretreated the colon cancer cells with STAT3 inhibitor stattic (50 μM) or NF-κB inhibitor PAO (0.1 μM) for 1 h, and then these cells were treated with (E)-4-(3-(3,5-dimethoxyphenyl)allyl)-2-methoxyphenol (10 μg/ml) for 24 h. As a result, PAO magnified (E)-4-(3-(3,5-dimethoxyphenylallyl)-2-methoxyphenol-induced inhibition of cell growth (Figure 3C). Stattic magnified (E)-4-(3-(3,5-dimethoxyphenylallyl)-2-methoxyphenol-induced inhibition of cell growth (Figure 3D), increased the expression of death receptors more significantly (Figure 3E) and suppressed DNA binding activity of STAT3 more significantly (Figure 3F).

### Increased effect of STAT3 siRNA or p50 siRNA on (E)-4-(3-(3,5-dimethoxyphenyl)allyl)-2-methoxyphenol-induced cell growth inhibition

**Figure 4:**
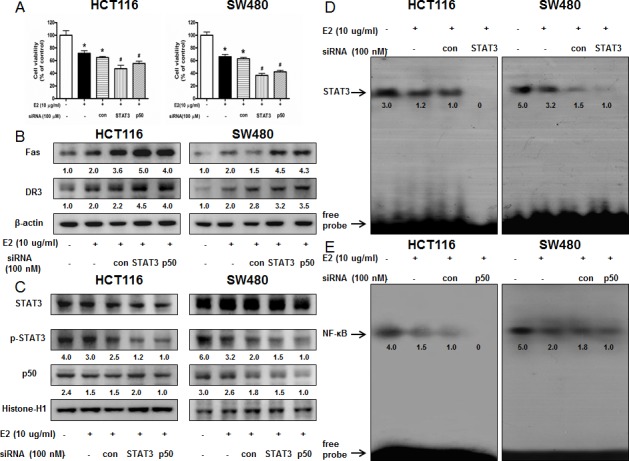
Effect of STAT3 siRNA or p50 siRNA on (E)-4-(3-(3,5-dimethoxyphenyl)allyl)-2-methoxyphenol-induced colon cancer cell growth and expression of DRs **A.** Colon cancer cells were treated with non-targeting control siRNA, STAT3 siRNA and p50 siRNA (100 nM) for 24 h, and then were treated with (E)-4-(3-(3,5-dimethoxyphenyl)allyl)-2-methoxyphenol (10 μg/ml) at 37°C for another 24 h. Relative cell survival rate was determined by MTT assay. Data was expressed as the mean ± S.D. of three experiments. *p < 0.05 indicates significant difference from control cells. #*p* < 0.05 indicates significant difference from (E)-4-(3-(3,5-dimethoxyphenyl)allyl)-2-methoxyphenol treated cells. **B.** Effect of STAT3 siRNA and p50 siRNA on the expression of regulatory proteins was determined by using Western blot analysis with antibodies against Fas, DR3 and β-actin (internal control). Each band is representative for three experiments. **C.** Nuclear proteins were used to determine expression of STAT3, p-STAT3, p50 and Histone H1 (internal control) in colon cancer cells. Each band is representative for three experiments. **D.** Effect of STAT3 siRNA on the DNA binding activity of STAT3. **E.** Effect of p50 siRNA on the DNA binding activity of NF-κB. Nuclear extract was incubated in binding reactions of ³²p-end-labeled oligo nucleotide containing the STAT3 or NF-κB sequence. The present EMSA results are representative for three experiments.

To determine the relationship between STAT3 or NF-κB activity and colon cancer cell growth inhibitory effect of (E)-4-(3-(3,5-dimethoxyphenyl)allyl)-2-methoxyphenol, we transfected the cells with STAT3 siRNA or p50 siRNA using a transfection agent. The cells were transfected with 100 nM STAT3 siRNA or p50 siRNA for 24 h, and then treated with (E)-4-(3-(3,5-dimethoxyphenyl)allyl)-2-methoxyphenol (10 μg/ml) for 24 h. Knock down of STAT3 or p50 magnified the cell growth inhibitory effect of (E)-4-(3-(3,5-dimethoxyphenyl)allyl)-2-methoxyphenol in HCT116 and SW480 colon cancer cells (Figure 4A). Knock down of STAT3 or p50 magnified the expression of Fas and DR3 (Figure 4B). The nucleus expression of p-STAT3 was decreased more significantly with knock down of STAT3 with STAT3 siRNA and the nucleus expression of p50 was decreased more significantly with knock down of p50 with p50 siRNA. Knock down of STAT3 with STAT3 siRNA inhibited the DNA binding activity of STAT3 more significantly (Figure 4D) and knock down of p50 with p50 siRNA inhibited the DNA binding activity of NF-κB more significantly (Figure 4E).

### Structure of (E)-4-(3-(3,5-dimethoxyphenyl)allyl)-2-methoxyphenol and binding between (E)-4-(3-(3,5-dimethoxyphenyl)allyl)-2-methoxyphenol and STAT3 as well as NF-κB

**Figure 5:**
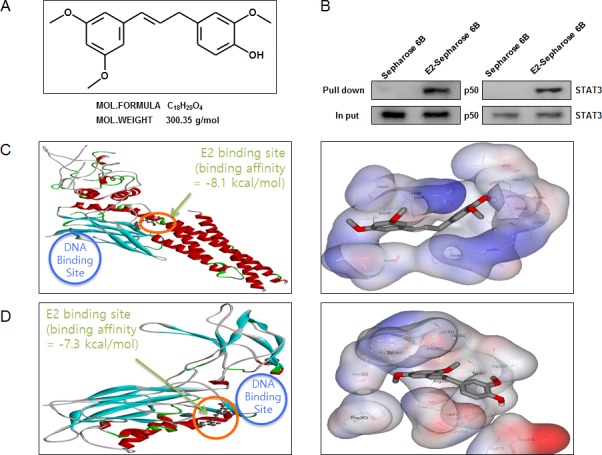
Structure of (E)-4-(3-(3,5-dimethoxyphenyl)allyl)-2-methoxyphenol, and binding between (E)-4-(3-(3,5-dimethoxyphenyl)allyl)-2-methoxyphenol and STAT3 as well as NF-κB **A.** Structure of (E)-4-(3-(3,5-dimethoxyphenyl)allyl)-2-methoxyphenol. **B.** Pull-down assay identifies a binding between (E)-4-(3-(3,5-dimethoxyphenyl)allyl)-2-methoxyphenol and STAT3 as well as NF-κB. (E)-4-(3-(3,5-dimethoxyphenyl)allyl)-2-methoxyphenol was conjugated with epoxy-activated Sepharose 6B. **C.** Docking model of (E)-4-(3-(3,5-dimethoxyphenyl)allyl)-2-methoxyphenol with STAT3 as described in methods. **D.** Docking model of (E)-4-(3-(3,5-dimethoxyphenyl)allyl)-2-methoxyphenol with NF-κB p50 subunit as described in methods.

The structure of (E)-4-(3-(3,5-dimethoxyphenyl)allyl)-2-methoxyphenol was showed by MedChem Designer 3.0 (Figure 5A). The binding between (E)-4-(3-(3,5-dimethoxyphenyl)allyl)-2-methoxyphenol and STAT3 as well as NF-κB p50 subunit was assessed by pull-down assay. The binding of (E)-4-(3-(3,5-dimethoxyphenyl)allyl)-2-methoxyphenol-Sepharose 6B beads with STAT3 and p50 was then detected by immunoblotting with STAT3 antibody and p50 antibody. The results indicated that (E)-4-(3-(3,5-dimethoxyphenyl)allyl)-2-methoxyphenol bound with cell lysates containing STAT3 and p50 from HCT116 cells (Figure 5B). To identify the binding site of (E)-4-(3-(3,5-dimethoxyphenyl)allyl)-2-methoxyphenol to STAT3 and p50, we performed computational docking experiments with (E)-4-(3-(3,5-dimethoxyphenyl)allyl)-2-methoxyphenol and STAT3 (Figure 5C) as well as p50 (Figure 5D).

### Effect of (E)-4-(3-(3,5-dimethoxyphenyl)allyl)-2-methoxyphenol on colon cancer tumor growth

**Figure 6:**
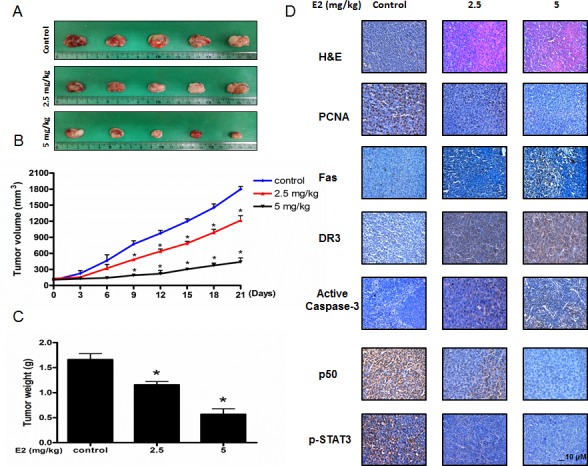
Anti-tumor activity of (E)-4-(3-(3,5-dimethoxyphenyl)allyl)-2-methoxyphenol in colon cancer xenograft **A.**, **B.** & **C.** Growth inhibition of subcutaneously transplanted HCT16 xenografts mice treated with MMPP (2.5 mg/kg and 5 mg/kg twice a week) for 3 weeks. Xenograft mice (*n* = 10) were administrated intraperitoneally with 0.01% DMSO or (E)-4-(3-(3,5-dimethoxyphenyl)allyl)-2-methoxyphenol (2.5 mg/kg and 5 mg/kg). Tumor burden was measured once per week using a caliper, and calculated volume length (mm) × width (mm) × height (mm)/2. Tumor weight and volume are presented as means ± S.D. **D.** Immunohistochemistry was used to determine expression levels of H&E, PCNA, Fas, DR3, active caspase-3, p-STAT3, p50 in nude mice xenograft tissues by the different treatments as described in the Materials and Methods section. Bar indicates 10 μm.

To elucidate the anti-tumor effect of (E)-4-(3-(3,5-dimethoxyphenyl)allyl)-2-methoxyphenol *in vivo*, the tumor growth on colon cancer cell xenograft bearing nude mice following (E)-4-(3-(3,5-dimethoxyphenyl)allyl)-2-methoxyphenol treatments was investigated. In HCT116 xenograft studies, (E)-4-(3-(3,5-dimethoxyphenyl)allyl)-2-methoxyphenol (2.5 mg/kg and 5 mg/kg) was administrated intraperitoneally twice per week for 3 weeks to mice which have tumors ranging from 100-150 mm^3^. Tumor volume was measured twice a week, and all mice were sacrificed at the end of experiment when tumors were dissected and weighted. The inhibitory effect of (E)-4-(3-(3,5-dimethoxyphenyl)allyl)-2-methoxyphenol on the growth of colon tumor was significant in xenograft mice model (Figure 6A). Tumor volume and weight were dose-dependently decreased (Figure 6B & Figure 6C). The immunohistochemistry analysis of tumor section by hematoxylin and eosin staining and proliferation antigens against PCNA staining revealed that (E)-4-(3-(3,5-dimethoxyphenyl)allyl)-2-methoxyphenol dose-dependently inhibited tumor growth, and the expression level of Fas, DR3, active caspase-3 was increased while the expression level of phosphor-STAT3 and p50 was decreased in nude mice xenograft tissues (Figure 6D).

### Effect of (E)-4-(3-(3,5-dimethoxyphenyl)allyl)-2-methoxyphenol on the expression of apoptosis regulatory proteins, as well as the DNA binding activity of NF-κB and STAT3 in colon tumor tissues

**Figure 7:**
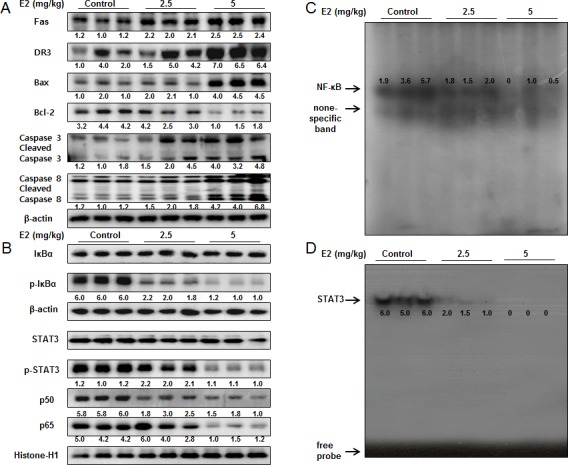
Effect of (E)-4-(3-(3,5-dimethoxyphenyl)allyl)-2-methoxyphenol on the expression of apoptosis regulatory proteins and the DNA binding activity of STAT3 and NF-κB in colon cancer tumors **A.** Expression of apoptosis regulatory proteins was determined by Western blot analysis with antibodies against Fas, DR3, capase-3, caspase-8, Bax, Bcl-2 and β-actin (internal control). **B.** Cytosol extracted proteins were used to determine the expression of IκBα, p-IκBα and β-actin (internal control). Nuclear extracted proteins were used to determine the expression of p50, p65, STAT3, p-STAT3 and Histone H1 (internal control) in colon cancer tumors. Each band is representative for three experiments. **C.** & **D.** Tumors were lysed with A buffer and C buffer. Nuclear extracts were incubated in binding reactions of ³²p-end-labeled oligo nucleotide containing the STAT3 or NF-κB sequence. The present EMSA results are representative for three experiments.

To examine the relationship between colon tumor growth and apoptosis regulatory proteins, we performed Western blotting assay. We found the expression of Fas, DR3, cleaved caspase-3, cleaved caspase-8 and Bax was increased while the expression of Bcl-2 was decreased in a dose dependent manner (2.5 mg/kg-5 mg/kg) (Figure 7A). The nucleus expression of p-STAT3, p-IκBα, p50 and p65 was decreased in a dose dependent manner (2.5 mg/kg-5 mg/kg) (Figure 7B). We also found the DNA binding activities of NF-κB (Figure 7C) and STAT3 (Figure 7D) were decreased in a dose dependent manner (2.5 mg/kg-5 mg/kg).

## DISCUSSION

In this study, we prepared (E)-4-(3-(3,5-dimethoxyphenyl)allyl)-2-methoxyphenol without the unstable aldehyde functionality by Heck reaction. Our findings showed that STAT3 binding affinity of (E)-4-(3-(3,5-dimethoxyphenyl)allyl)-2-methoxyphenol was −8.1 kcal/mol, p50 binding affinity was −7.3 kcal/mol, the IC_50_ of STAT3 luciferase activities was 3.1 μg/ml. The previous data showed that cancer cell growth was inhibited by treatment with (E)-2,4-Bis(*p*-hydroxyphenyl)-2-butenal in SW620 and HCT116 colon cancer cells with IC_50_ values 27 μg/ml and 25 μg/ml, respectively [24]. In contrast, cancer cell growth was inhibited by treatment with (E)-4-(3-(3,5-dimethoxyphenyl)allyl)-2-methoxyphenol in SW480 and HCT116 colon cancer cells with IC_50_ values 12.3 μg/ml and 15.7 μg/ml, respectively. These data indicated that (E)-4-(3-(3,5-dimethoxyphenyl)allyl)-2-methoxyphenol inhibited colon cancer cell growth with much lower doses in comparison to (E)-2,4-Bis(*p*-hydroxyphenyl)-2-butenal.

In the present study, we found that (E)-4-(3-(3,5-dimethoxyphenyl)allyl)-2-methoxyphenol treatment increased the expression of apoptotic proteins such as Bax, cleaved caspase-3, cleaved caspase-8 as well as the expression of death receptors like DR3 and Fas in a concentration dependent manner. However, the expression of anti-apoptotic protein Bcl-2 was decreased. Several anti-apoptotic proteins, such as Survivin and members of the Bcl family (Bcl-xl, Bcl-2 and Mcl-1) which are known to be crucial for tumor cell survival, are direct target genes of STAT3 and are down-regulated as a consequence of STAT3 inhibition [25]. Several studies have proposed that STAT3 signaling may be involved in colorectal carcinogenesis [26, 27]. Besides, inhibition of JAK1,2/STAT3 signaling induces apoptosis, cell cycle arrest, reduces tumor cell invasion in colorectal cancer cells [28]. Ursolic acid inhibits the growth of colon cancer-initiating cells by inhibition of STAT3 [29]. CAY10598 induced apoptosis in HCT116 cells through the generation of ROS and inactivation of JAK2/STAT3 signaling [30]. Expression of p-STAT3 was immunohistochemically examined and was found 57.4% in 108 cases of colorectal adenocarcinoma tissue obtained at surgey [27]. In agreement with this notion, our present data showed that (E)-4-(3-(3,5-dimethoxyphenyl)allyl)-2-methoxyphenol suppressed DNA binding activity of STAT3. The present data also showed that (E)-4-(3-(3,5-dimethoxyphenyl)allyl)-2-methoxyphenol directly bound to STAT3, and then blocked the activity of STAT3. Besides, docking model exactly showed that (E)-4-(3-(3,5-dimethoxyphenyl)allyl)-2-methoxyphenol bound in between coiled coil domain (CCD) and DNA binding domain (DBD) of STAT3 (bound inside a flight pocket comprised of Ala241, Lys244, Arg245, Gln247, Val322, Val323, Gln326, Leu453, Thr456, His457, Asn485 and Pro487). Moreover, (E)-4-(3-(3,5-dimethoxyphenyl)allyl)-2-methoxyphenol-induced inhibitory effect of cancer cell growth was magnified by knock down of STAT3 with STAT3 siRNA. These data indicate that STAT3 plays a critical role in (E)-4-(3-(3,5-dimethoxyphenyl)allyl)-2-methoxyphenol-induced anti-cancer effect in human colon cancer cells.

Besides, NF-κB is also involved in growth arrest and apoptosis by suppressing the expression of various target genes such as Bax, caspase-3, caspage-9 and activating Bcl-2 and Survivin [31, 32]. Increased nuclear translocation of NF-κB was found in colorectal carcinoma patients and it was demonstrated that the activation of NF-κB was correlated with tumor progression [33]. Tumor-associated macrophages from p50 deficient mice regained a proinflammatory (M1) phenotype associated with reduced tumor growth [34]. Blocking NF-κB can cause tumor cells to cease proliferation or become more sensitive to the action of antitumor agents [35]. Several agents have shown their anti-cancer activity through inhibition of NF-κB. In our previous study, we found that inflexinol inhibited colon cancer cell growth through inhibition of NF-κB via direct binding to NF-κB p50 subunit [36]. In this study, our data also showed that (E)-4-(3-(3,5-dimethoxyphenyl)allyl)-2-methoxyphenol suppressed DNA binding activity of NF-κB. The decrease of NF-κB DNA binding activity was associated with the inhibitory effect of (E)-4-(3-(3,5-dimethoxyphenyl)allyl)-2-methoxyphenol on the IκB phosphorylation and nuclear translocation of NF-κB p50 and p65 subunits. Moreover, co-treatment with NF-κB inhibitor, PAO (0.1 μM) and (E)-4-(3-(3,5-dimethoxyphenyl)allyl)-2-methoxyphenol (10 μg/ml) inhibited colon cancer cell growth more significantly. However, deletion of p50 by siRNA magnified (E)-4-(3-(3,5-dimethoxyphenyl)allyl)-2-methoxyphenol-induced inhibitory effect on colon cancer cell growth. In addition, docking model showed that (E)-4-(3-(3,5-dimethoxyphenyl)allyl)-2-methoxyphenol bound near to the DNA binding site (in a pocket created by Phe353, Tyr357, Val358, Glu360, Gly361, Pro362, Ser363, His364, Gly365, Val412, Cys416, Asp418 and Leu440). Thus, it is possible that (E)-4-(3-(3,5-dimethoxyphenyl)allyl)-2-methoxyphenol inhibits colon cancer cell growth via inhibition of both STAT3 and NF-κB pathways. Activated STAT3 in cancer cells can ensure constitutive NF-κB activation, even when the IKK complex is only temporarily activated [38]. Despite their potential side effects, inhibition of STAT3 and NF-κB may represent a good approach to combat cancer [23]. Studies have also shown that constitutive STAT3 activation is associated with the inhibition of death receptor mediated apoptosis [39]. Similarly, NF-κB directly inhibits Fas transcription to suppress Fas-mediated apoptosis and tumor suppression [40]. STAT3 and NF-κB cooperate to promote the development and progression of colon cancer [23], and thus prevent cell death through inhibition of the extrinsic apoptotic pathway which is activated upon binding of extracellular ligands to cell-surface DRs [39].

It was also reported that several promising anti-cancer compounds induced apoptotic cell death through activation of DRs. The snake venom toxin from *Vipera lebetina turanica* induced the apoptosis of colon cancer cells through reactive oxygen species (ROS) and c-Jun N-terminal kinases (JNK) dependent death receptor (DR4 and DR5) expression [41]. Garcinol, a polyisoprenylated benzophenone derivative, derived from dried rind of the fruit Garcinia indica can potentiated TRAIL-induced apoptotic cell death of human colon cancer cell through up-regulation of DR4 and DR5 [42]. Zerumbone enhanced TRAIL-induced apoptosis through the induction of death receptors in human colon cancer cells [43]. Similarly, our findings showed that (E)-4-(3-(3,5-dimethoxyphenyl)allyl)-2-methoxyphenol induced apoptotic cell death through activation of Fas and DR3 in colon cancer cell lines. Moreover, the expression of Fas and DR3 was increased more significantly in STAT3 siRNA or p50 siRNA transfected cells. Similarly, co-treatment with (E)-4-(3-(3,5-dimethoxyphenyl)allyl)-2-methoxyphenol and STAT3 inhibitor Stattic (50 μM) or PAO (0.1 μM) increased the expression of Fas and DR3 more significantly. In addition, we also evaluated the anti-cancer effect of (E)-4-(3-(3,5-dimethoxyphenyl)allyl)-2-methoxyphenol on colon tumor growth. As a result, (E)-4-(3-(3,5-dimethoxyphenyl)allyl)-2-methoxyphenol inhibited colon tumor growth in a dose dependent manner (2.5 mg/kg-5 mg/kg). And the expression level of Fas, DR3, cleaved caspase-3, cleaved caspase-8 and Bax was increased while the expression of Bcl-2 was decreased in a dose dependent manner (2.5 mg/kg-5 mg/kg). Moreover, the DNA binding activities of both STAT3 and NF-κB were suppressed in a dose dependent manner (2.5 mg/kg-5 mg/kg).

In conclusion, (E)-4-(3-(3,5-dimethoxyphenyl)allyl)-2-methoxyphenol could inhibit cell growth of colon cancer *in vivo* and *in vitro* through activation of Fas, DR3 and inhibition of STAT3 and NF-κB pathways. Therefore, (E)-4-(3-(3,5-dimethoxyphenyl)allyl)-2-methoxyphenol may be a promising anti-cancer agent for treatment of colon cancer.

## MATERIALS AND METHODS

### Chemicals

Heck reaction was used for the synthesis starting from phenyl halide moieties with substituents (2.0 mmol) and allylbenzene moieties with substituents (2.0 mmol). Phenyl halide (2.0 mmol) and allylbenzene (2.0 mmol) were added with triphenylphosphine (105 mg, 0.4 mmol), Pd(OAc)_2_ (44.9 mg, 0.2 mmol), and tributylamine (451 mg, 1.9 mmol) in a 25 ml round bottom flask and the reaction mixture was stirred for 2 h at 45°C under argon atmosphere. The product was purified by flash silica gel chromatography using hexane and ethyl acetate (3:1 mixture v/v) as the mobile phase.

### Materials

Caspase-3, caspase-8 antibodies were purchased from Cell Signaling Technology Inc. (Beverly, MA). Fas, DR3, DR4, DR5, FasL, TWEAK, TRAIL, p50, p65, IκBα, phospho-IκBα, STAT3, phospho-STAT3, Bcl-2, Bax, Histone-H1 and β-actin antibodies were purchased from Santa Cruz Biotechnology, Inc. (Santa Cruz, CA). The cell culture materials were obtained from GIBGO^®^ of Introgen™ (Seoul, Korea), and other chemical reagents were from Sigma Chemical Co.

### Cell culture

The HCT116, SW480 colon cancer cell lines and CCD-18Co colon epithelial normal cell line were obtained from American Type Culture Collection (Manassas, VA, USA). HCT116 was cultured in DMEM (Gibco, Life Technologies, Grand Island, NY) medium supplemented with 10% heat inactivated fetal bovine serum (FBS) and 100 units/ml penicillin, 100 μg/ml streptomycin. SW480 was cultured in RPMI 1640 medium supplemented with 10% heat inactivated FBS and 100 units/ml penicillin, 100 μg/ml streptomycin. CCD-18Co was cultured in DMEM medium supplemented with 10% heat inactivated FBS, 100 units/ml penicillin, 100 μg/ml streptomycin and 0.1 mM non-essential amino acids. Cell cultures were then maintained in an incubator within a humidified atmosphere of 5% CO_2_ at 37°C.

### Cell viability assay

Colon cancer cells HCT116 and SW480 were cultured in 96-well plates for 24 h, then were treated with (E)-4-(3-(3,5-dimethoxyphenyl)allyl)-2-methoxyphenol (0- 20 μg/ml) for 24 h. After treatment, cell viability was measured by MTT [3-(4, 5-Dimethylthiazol-2-yl)-2, 5-Diphenyltetrazolium Bromide] assay (Sigma Aldrich, St. Louis, MO) according to the manufacturer's instructions. Briefly, MTT (5 mg/ml) was added to cells and plates were incubated at 37°C for 2-4 h before dimethyl sulfoxide (100 μl) was added to each well. Finally, the absorbance of each well was read at a wavelength of 540 nm using a plate reader.

### Apoptosis evaluation

Colon cancer cells HCT116 and SW480 were cultured on 8-chamber slides for 24 h and then were treated with (E)-4-(3-(3,5-dimethoxyphenyl)allyl)-2-methoxyphenol (0-15 μg/ml) for 24 h. TUNEL assays were performed by using the DeadEnd™ Fluorometric TUNEL System (Promega Corporation, Madison, USA) according to manufacturer's instructions. Total number of cells in a given area was determined by using DAPI (Vector Laboratories, Inc., Burlingame, CA) staining. The cells were then observed through a fluorescence microscope (Leica Microsystems AG, Wetzlar, Germany). The apoptotic index was determined as the number of TUNEL-positive stained cells divided by the total cell number counted x100%.

### Western blotting

Colon cancer cells treated with (E)-4-(3-(3,5-dimethoxyphenyl)allyl)-2-methoxyphenol (0-15 μg/ml) for 24 h were homogenized with a protein extraction solution (PRO-PREP^TM^, Intron Biotechnology), and lysed for 60 minutes incubation on ice. The cell lysate was centrifuged at 13,000 rpm for 15 minutes at 4°C. Equal amount of proteins (40 μg) were separated on a SDS/12%-polyacrylamide gel, and then transferred to a polyvinylidene fluoride (PVDF) membrane (GE Water and Process technologies, Trevose, PA, USA). Blots were blocked for 1 h at room temperature with 5% (w/v) non-fat dried milk in Tris-Buffered Saline Tween-20 [TBST: 10 mM Tris (pH 8.0) and 150 mM NaCl solution containing 0.05% Tween-20]. After a short washing in TBST, the membranes were immunoblotted with the following primary antibodies: caspase-3, caspase-8 (1:1000 dilutions; Cell Signaling, Beverly, MA) and Fas, DR3, p50, p65, STAT3, phospho-STAT3, Bcl-2, Bax, Histone-H1 and β-actin (1:1000 dilutions; Santa Cruz Biotechnology, Santa Cruz, CA). The blots were performed using specific antibodies followed by second antibodies and visualization by chemiluminescence (ECL) detection system.

### Electrophoretic mobility shift assay

The DNA binding activity of STAT3 was determined using EMSA according to the manufacturer's recommendations (Promega). In short, HCT116 and SW480 cells were cultured on 100-mm culture dishes. After treatment with (E)-4-(3-(3,5-dimethoxyphenyl)allyl)-2-methoxyphenol for 2 h, the cells were washed twice with PBS, followed by the addition of 1 ml of phosphate buffered saline (PBS), and the cells were scraped into a cold eppendorf tube. The cells were lysed in ice-cold buffer A (10 mM HEPES, 1.5 mM MgCl_2_, 10 mM KCl, 0.5 mM DTT, 0.2 mM PMSF, 0.1% protase inhibitor, 0.1% phosphatase inhibitor and 0.5% NP40) for 30 minutes, and centrifuged for 6 minutes at 6,000 rpm. The residual pellet was resuspended in buffer C (10 mM HEPES, 1.5 mM MgCl_2_, 0.5 mM DTT, 0.2 mM PMSF, 0.1% protase inhibitor, 0.1% phosphatase inhibitor, 420 mM NaCl, 0.2 mM EDTA and 20% glycerol). After incubation at 4°C for 1 h, the lysate was centrifuged for 15 minutes at 13,000 rpm and then nuclear extracts were prepared and processed for EMSA as previously described. The relative densities of the DNA-protein binding bands were scanned by densitometry using MyImage (SLB), and quantified by Labworks 4.0 software (UVP, Inc., Upland, CA).

### Transfection of siRNA

Colon cancer cells were plated in 6-well plates (2 × 10^5^ cells / well) and were transiently transfected with siRNA, using a mixture of siRNA and the WellFect-EX PLUS reagent in OPTI-MEM, according to the manufacturer's specification (WelGENE, Seoul, Korea). The transfected cells were treated with (E)-4-(3-(3,5-dimethoxyphenyl)allyl)-2-methoxyphenol (10 μg/ml) for 24 h and then used for detecting cell viability and protein expression.

### Pull-down assay

(E)-4-(3-(3,5-dimethoxyphenyl)allyl)-2-methoxyphenol was conjugated with epoxy-activated Sepharose-6B. (E)-4-(3-(3,5-dimethoxyphenyl)allyl)-2-methoxyphenol (1 mg) was dissolved in 1ml of coupling buffer (0.1 M NaHCO_3,_ pH 11.0 containing 0.5 M NaCl). The epoxy-activated Sepharose 6B was swelled and washed in distilled water on a sintered glass filter, then washed with the coupling buffer. Epoxy-activated Sepharose 6B beads were added to the (E)-4-(3-(3,5-dimethoxyphenyl)allyl)-2-methoxyphenol-containing coupling buffer and rotated at 4°C for overnight. After washing, unoccupied binding sites were blocked with 0.1 M Tris-HCl buffer (pH 8.0) 2 h at room temperature. The (E)-4-(3-(3,5-dimethoxyphenyl)allyl)-2-methoxyphenol-conjugated Sepharose 6B was washed with three cycles of alternating pH wash buffers (buffer I: 0.1 M acetate and 0.5 M NaCl, pH 4.0; buffer II: 0.1 M Tris-HCl and 0.5 M NaCl, pH 8.0). The control unconjugated epoxy-activated Sepharose 6B beads were prepared as described above in the absence of (E)-4-(3-(3,5-dimethoxyphenyl)allyl)-2-methoxyphenol. The cell lysate was mixed with (E)-4-(3-(3,5-dimethoxyphenyl)allyl)-2-methoxyphenol-conjugated Sepharose 6B or Sepharose 6B at 4°C for overnight. The beads were then washed on time with TBST. The bound proteins were eluted with SDS loading buffer. The proteins were then resolved by SDS-PAGE followed by immunoblotting with antibodies against STAT3 and p50 (1:1000 dilutions, Santa Cruz Biotechnology, Santa Cruz, CA).

### Docking experiment

Docking studies between STAT3 or p50 and (E)-4-(3-(3,5-dimethoxyphenyl)allyl)-2-methoxyphenol was performed using Autodock VINA [44]. Only one monomer of the homo-dimeric STAT3 or p50 crystal structure was used in the docking experiment and conditioned using AutodockTools by adding all polar hydrogen atoms. Three dimensional structures of STAT3-DNA complexes and p50-DNA complexes were retrieved from the Protein Data Bank (PDB codes: STAT3-3CWG, p50-1VKX). Starting from the co-crystallized complexes, the STAT3 or p50 monomer chain, (E)-4-(3-(3,5-dimethoxyphenyl)allyl)-2-methoxyphenol for docking were prepared using Maestro graphical interface. The grid box was centered on the STAT3 or p50 monomer and the size of the grid box was adjusted to include the whole monomer. Docking experiments were performed at various exhaustiveness values of the defaults: 16, 24, 32, 40 and 60. Molecular graphics for the best binding model was generated using Discovery Studio Visualizer 2.0.

### Antitumor activity study in *in vivo* xenograft animal model

Eight-week-old male BALB/C nude mice were purchased from Orient-Bio (Gyunggi-do, Korea). The mice were maintained in accordance with the Korea Food and Drug Administration guidelines as well as the regulations for the care and use of laboratory animals of the animal ethics committee of Chungbuk National University (CBNU-278-11-01). Human colon cancer cell line HCT116 cells were injected subcutaneous (1 × 10^7^ cells/0.1 ml PBS/animals) with a 27-gauge needle into the right lower flanks in carrier mice. After 14 days, when the tumors had reached an average volume of 100-150 mm^3^, the tumor-bearing nude mice were intraperitoneally (i.p.) injected with (E)-4-(3-(3,5-dimethoxyphenyl)allyl)-2-methoxyphenol (2.5 mg/kg and 5 mg/kg dissolved in 0.01% DMSO) twice per week for 3 weeks. The group treated with 0.01% DMSO was designed as the control. The weight and tumor volume of the animals were monitored twice per week. The tumor volumes were measured with vernier calipers and calculated by the following formula: (A × B^2^)/2, where A is the larger and B is the smaller of the two dimensions. At the end of the experiment, the animals were sacrificed. The tumors were separated from the surrounding muscles and dermis, excised and weighed.

### Immunohistochemistry

The animal tissues were fixed in 4% paraformaldehyde and cut into 10 μm sections using a freezing microtome (Thermo Scientific, Germany). The sections were stained with hematoxylin and eosin (H&E) for pathological examination. For immunohistological staining, tumor sections were incubated with primary antibody against PCNA, Fas, DR3, active caspase-3, phospho-STAT3, p50 (1:500, Abcam, Cambridge, UK). After rinse in phosphate buffered saline (PBS), the sections were subject to incubation in biotinylated secondary antibody. The tissue was incubated for 1 h in an avidin-peroxidase complex (ABC, Vector Laboratories, Inc., Burlingame, CA). After washing in PBS, the immunocomplex was visualized using 3, 3-diaminobenzidine solution (2 mg/10 ml) containing 0.08% hydrogen peroxide in PBS. Sections were dehydrated in a series of graded alcohols, cleared in xylene and coverslipped using Permount (Fisher Scientific, Suwanee, GA).

### Statistical analysis

The data was analyzed by GraphPad Prism 4 software (Version 4.03, GraphPad Software, La Jolla, CA). Data was presented as mean ± S.D. The differences in all data were assessed by one-way analysis of variance. When the *p* value in the ANOVA test indicated statistical significance, the differences were assessed by the Dunnet's test. A value of *p* < 0.05 was considered to be statistically significant.
